# Different Types of Physical Activity and Metabolic Control in People With Type 1 Diabetes Mellitus

**DOI:** 10.3389/fphys.2019.01210

**Published:** 2019-09-24

**Authors:** Iztok Štotl, Tim Kambič, Vedran Hadžić, Anže Zdolšek

**Affiliations:** ^1^Department of Endocrinology, Diabetes and Metabolic Diseases, University Medical Centre Ljubljana, Ljubljana, Slovenia; ^2^Faculty of Sport, University of Ljubljana, Ljubljana, Slovenia; ^3^Department for Research and Education, General Hospital Murska Sobota, Murska Sobota, Slovenia

**Keywords:** physical activity, type 1 diabetes, obesity, hypoglycemia, metabolism

## Abstract

**Background:**

The aim of presented cross-sectional study was to determine the association of different types of physical activity (PA) with metabolic control in people with type 1 diabetes.

**Materials and Methods:**

A total of 109 adult subjects with type 1 diabetes were asked to complete the non-exercise activity thermogenesis (NEAT) questionnaire, the hypoglycemia questionnaire, and the World Health Organization Global PA Questionnaire (GPAQ) which was used to assess moderate PA (MPA) and vigorous PA (VPA).

**Results:**

NEAT score (*p* < 0.001) and total duration of work as assessed with GPAQ (*p* = 0.007) were positively associated with chronic glycemic control when controlled for sex, BMI, and continuous glucose monitoring system (CGMS) use. We could not confirm such association with total leisure time PA (LTPA) assessed with GPAQ (*p* = 0.443), though. Multivariate regression model controlled for sex showed positive effects of HbA_1__*c*_ (*p* = 0.011) and age (*p* = 0.035), and negative effect of NEAT score (*p* = 0.001) on BMI. Systolic blood pressure was positively associated with duration of MPA (*p* = 0.009) and VPA (*p* = 0.012), but not with NEAT score (*p* = 0.830) when controlled for sex and BMI. NEAT score and VPA were positively associated with HDL levels when controlled for sex and BMI. Controlled for sex and BMI, higher values of VPA were significantly associated with lower levels of total cholesterol (*p* = 0.009) and LDL (*p* = 0.005).

**Conclusion:**

Higher levels of NEAT are associated with some favorable metabolic effects in adult people with type 1 diabetes, but may also present an additional burden for them with more challenging environment regarding glycemic control.

## Introduction

Physical activity has an important role in many aspects of treatment of people with type 1 diabetes (Riddell et al., 2017). Recent meta-analysis did not show benefit of exercise as measured by HbA_1__*c*_ (Ostman et al., 2018), while other studies show inconsistent results (Chimen et al., 2012). Large cross-sectional study of 18,028 adults with type 1 diabetes showed that people who engaged more in recreational exercise had better HbA_1__*c*_ concentrations, a more favorable BMI, less dyslipidemia and hypertension, and fewer diabetes-related complications (retinopathy and microalbuminuria) than those who were less habitually active (Bohn et al., 2015). A 7-year prospective cohort analysis performed in 1,659 people with type 1 diabetes could not find association between HbA_1__*c*_ levels and amount of LTPA (Balk et al., 2016). In another cross-sectional study of 1,030 people with type 1 diabetes low levels of LTPA were associated with poor glycemic control in women, but there was no statistical association with levels of LTPA and HbA_1__*c*_ in men (Wadén et al., 2005).

Physical activity can be divided into structured PA and NEPA. NEPA is distinct from purposeful exercise and includes every PA that is not sleeping, eating, or sports-like exercise and includes various activities in daily life such as going to work, attending school, singing, dancing, washing clothes, cleaning floor, etc. NEAT represents the energy expenditure associated with these activities (Levine, 2004). The variety of different types of activity that composes total NEPA creates a challenge for assessment of NEAT. Researchers have tried to estimate NEPA/NEAT with the use of double labeled water, indirect calorimetry, accelerometer, pedometer, inclinometer, heart rate, and activity diary (Silva et al., 2018).

Amount of NEPA affects daily glucose excursions (Ogata et al., 2013). Interrupting sitting time with short bouts of light or moderate intensity walking lowers postprandial glucose and insulin levels in overweight/obese adults (Dunstan et al., 2012). In people with type 1 diabetes, slow-pace walking after a meal improves postprandial glucose excursions when observed in controlled study environment (Manohar et al., 2012). Routine daily PAs of everyday life seem to be coupled with glucose variations in type 1 diabetes (Farabi et al., 2015). Recent study has successfully provided quantitative information on correlation between mild PA and short-term glucose dynamics in type 1 diabetes (Zecchin et al., 2013), that could be used in future models to improve glucose management with combination of advanced sensor technologies (Ding and Schumacher, 2016).

Majority of research that tries to study effects of PA on metabolic control in people with type 1 diabetes assesses predominantly planned exercise or LTPA. While higher levels of NEAT are known to be associated with many health benefits in people with type 2 or without diabetes (Levine, 2015), long-term metabolic effects of NEAT in people with type 1 diabetes have not been elucidated, yet. The aim of this study was to determine the association between different types of PA and metabolic control in people with type 1 diabetes.

## Materials and Methods

### Sample and Study Protocol

One hundred and nine adult people with documented type 1 diabetes, that agreed to participate in the study and signed a written consent, were consecutively enrolled in the study on their regular visit at outpatient diabetes clinic in University Medical Centre Ljubljana (from January to March 2018). People with substantial limitations for everyday PA or pregnancy were excluded from the study. One homeless person was also excluded, because his answers would be inappropriate for NEAT questionnaire in current form.

During the regular visits, body weight was measured using Seca 285 device (Seca GmbH, Germany), while blood pressure was obtained using automatic sphygmomanometer Omron M6 (Omron, Osaka, Japan). Blood samples were withdrawn for HbA_1__*c*_ analysis using D-100 Bio-Rad high-performance liquid chromatography method (Bio-Rad Laboratories, United States), whereas serum lipid profile was retrieved from electronic health record, if they were collected routinely within 1 year before inclusion (available for 75 participants). The study protocol was approved by the National Medical Ethics Committee of Slovenia (Ref. No. 0120-258/2017/4).

Participants were asked to complete World Health Organization GPAQ (Armstrong and Bull, 2006) for assessment of MPA and VPA at work (such as paid or unpaid work, study/training, household chores, harvesting food/crops, fishing or hunting for food, and seeking employment), during traveling to and from places and during recreational activities. Duration of VPA per week in minutes (VPA) was calculated with the sum of VPA at work and recreation, whereas duration of MPA per week in minutes (MPA) was calculated with the sum of MPA at work, recreation, and transport.

Non-exercise activity thermogenesis was assessed with the questionnaire that was developed by Hamasaki et al. (2013) for assessment of NEAT in people with type 2 diabetes. The NEAT questionnaire consists of 11 question items about daily locomotive activities (about commuting on foot, shopping for food, using stairs, etc.) and 25 question items about non-locomotive activities (being active in different domestic chores, looking after children, elderly, or pets, etc.). Prior to NEAT questionnaire administration, question regarding the karaoke singing was changed to choir singing, which is a more common habit in Slovenian population. Each questionnaire item was then evaluated with a score of one to three points in order of levels of habitual PA as described in original paper and added up to determine the total NEAT score. NEAT questionnaire tries to indirectly assess the amount of activity of daily living that is not always perceived as PA in other questionnaires for assessing PA. Its use for this task was validated by triaxial accelerometer in people with type 2 diabetes (Hamasaki et al., 2014). PA assessed by NEAT score partly overlaps with MPA assessed with GPAQ. Nevertheless, GPAQ has higher threshold for detecting PA while including only MPA that causes small increases in breathing or heart rate such as brisk walking (or carrying light loads) and also does not capture PA episodes shorter than 10 min.

Moreover, participants also reported the frequency of recent hypoglycemia. Frequency of hypoglycemia was defined with participant’s self-estimated number of documented hypoglycemias (<3.9 mmol/l) in the last month.

### Statistical Analysis

Data were analyzed using IBM SPSS 21 (IBM Inc., Chicago, IL, United States). Categorical data are presented as numbers (percentages), and numerical variables are presented as means (standard deviations). All numerical data were screened for normality of distribution using Shapiro–Wilk’s test and histograms. Correlations were calculated using Pearson’s correlation coefficient for normally distributed and linear correlation, otherwise Spearman’s rank correlation coefficient was applied. Additionally, the associations between demographic variables (sex, age, BMI), PA variables (NEAT, MPA, and VPA), and selected metabolic variables (HbA_1__*c*_, frequency of hypoglycemia), hemodynamics, and blood lipids were determined using the multivariate linear regression, after the data were screened for normality of distribution, homoscedasticity, linearity, and multicollinearity (Field, 2013). In the case of asymmetrically distributed outcome variable, the data were transformed using logarithmic (frequency of hypoglycemia, HDL) or 1/Y transformation (triglycerides, BMI, SBP). The significance level was set at *p*-values < 0.05.

## Results

A total of 109 people diagnosed with type 1 diabetes [54 men and 55 women, aged 38 (10) years, height 173.7 (8.7) cm, and weight 77.33 (15.70) kg] were included in the sample (Table 1), with mean type 1 diabetes duration of 22 (10) years. Majority of participants (65.1%) were regular users of insulin pump, 31% of them were also using CGMS. Thirty-five percent of the study participants used MDIs for glucose management, 13.2% of them used CGMS. Half of them failed to reach the goal for glycemic control [median HbA_1__*c*_ 53.0 mmol/mol (7.0%)] and reported more than four events (median 4.0 events per month) of hypoglycemia per month. In addition to therapy for glucose control, some participants had prescribed anti-hypertensive (15.0%) and/or medication therapy for dyslipidemia (14.0%). We observed statistically significant differences in PA between genders, with men spending more time for VPA and women being more active in NEPA. Correlations of different types of PA with different observed parameters of metabolic control are presented in Table 2 and multivariate regression models in Table 3.

**TABLE 1:** Baseline sample characteristics.

	**All**	**Men**	**Women**	***p*-value**	***N***
**Demographics**					
Gender, men/women (*n*)	109	54	55		109
Age (years)	38 (10)	37 (9)	38 (10)	0.849	108
Height (cm)	173.72 (8.74)	179.43 (7.17)	167.69 (5.67)	< 0.001	105
Weight (kg)	77.33 (15.70)	84.41 (13.04)	69.98 (14.93)	< 0.001	106
BMI (kg/m^2^)	25.54 (4.57)	26.22 (3.78)	24.83 (5.23)	0.008	105
**Diabetes characteristics**					
Insulin pump – *n* (%)	71 (65.1)	34 (63.0)	37 (67.3)	0.690	109
CGMS – *n* (%)	27 (24.8)	16 (29.6)	11 (20.0)	0.274	109
Duration of diabetes (years)	22 (10)	22 (11)	22 (10)	0.815	102
Frequency of hypoglycemia (*n*)	6 (4)	6 (4)	6 (5)	0.888	99
**Hemodynamics and blood markers**					
Systolic blood pressure (mmHg)	129.64 (17.18)	135.81 (16.43)	123.10 (15.41)	< 0.001	105
Diastolic blood pressure (mmHg)	79.10 (8.75)	81.17 (8.81)	76.90 (8.21)	0.011	105
HbA_1__c_ (mmol/mol)	53.28 (9.69)	53.86 (9.78)	52.69 (9.64)	0.346	106
HbA_1__c_ (%)	7.03 (0.89)	7.08 (0.90)	6.97 (0.88)	0.346	106
Serum cholesterol (mmol/L)	4.91 (0.73)	4.81 (0.66)	5.04 (0.80)	0.181	75
Serum HDL (mmol/L)	1.75 (0.50)	1.6 (0.41)	1.94 (0.54)	0.005	75
Serum LDL (mmol/L)	2.77 (0.62)	2.84 (0.64)	2.66 (0.58)	0.195	75
Triglycerides (mmol/L)	0.92 (0.45)	0.99 (0.56)	0.84 (0.25)	0.157	75
**Physical activity**					
MPA (min/week)	688.2 (711.13)	680.80 (806.86)	695.38 (612.85)	0.180	104
VPA (min/week)	418.16 (649.45)	501.47 (696.40)	336.44 (595.28)	0.039	103
NEAT score (points)	73.14 (9.85)	70.55 (10.15)	75.74 (8.90)	0.006	106

*BMI, body mass index; CGMS, continuous glucose monitoring system; HDL, high-density lipoprotein; LDL, low-density lipoprotein; MPA, moderate physical activity; VPA, vigorous physical activity; NEAT, non-exercise activity thermogenesis; data are expressed as mean (*SD*). *p* value < 0.05.*

**TABLE 2:** Correlations of PA types with clinical parameters and biomarkers.

			**All**		**Men**		**Women**	
**Parameter**	**PA**	***N***	***r***	***p*-value**	***r***	***p*-value**	***r***	***p*-value**
HbA_1c_	VPA	102	0.190	0.056	0.094	0.513	0.228	0.107
	MPA	103	0.044	0.660	−0.014	0.925	0.095	0.503
	NEAT	105	**0.226^‡^**	**0.021**	**0.286^‡^**	**0.038**	0.152	0.281

Hypoglycemia	VPA	96	−0.201	0.050	−0.224	0.118	−0.202	0.178
	MPA	96	−0.023	0.827	−0.055	0.708	−0.025	0.865
	NEAT	98	−0.165	0.105	−0.120	0.401	−0.276	0.060

SBP	VPA	101	**0.320**	**0.001**	0.211	0.137	**0.282**	**0.047**
	MPA	102	0.169	0.089	0.211	0.137	0.268	0.057
	NEAT	104	−0.150	0.127	**−0.273**^‡^	**0.048**	0.057	0.691

DBP	VPA	101	0.056	0.575	−0.016	0.914	0.048	0.740
	MPA	102	−0.017	0.866	0.007	0.960	0.033	0.819
	NEAT	104	−0.124^‡^	0.211	−0.116^‡^	0.408	−0.006^‡^	0.968

BMI	VPA	101	−0.008	0.934	0.002	0.988	−0.071	0.622
	MPA	102	−0.175	0.079	−0.112	0.431	−0.167	0.241
	NEAT	104	**−0.352**	**0.000**	**−0.411**	**0.002**	−0.202	0.154

Cholesterol	VPA	73	**−0.234**	**0.046**	−0.240	0.130	−0.150	0.412
	MPA	72	−0.123	0.303	−0.093	0.575	−0.144	0.424
	NEAT	74	−0.203^‡^	0.082	−0.278^‡^	0.078	−0.252^‡^	0.157

LDL	VPA	73	**−0.249**	**0.034**	**−0.344**	**0.028**	−0.261	0.149
	MPA	72	−0.111	0.354	−0.104	0.530	−0.047	0.796
	NEAT	74	−0.221^‡^	0.058	−0.228^‡^	0.151	−0.128^‡^	0.478

HDL	VPA	72	−0.071	0.550	0.158	0.324	−0.093	0.612
	MPA	71	−0.062	0.606	−0.083	0.613	−0.148	0.413
	NEAT	73	−0.112	0.341	−0.158^‡^	0.323	−0.328^‡^	0.062

Triglycerides	VPA	73	−0.036	0.764	−0.077	0.632	0.029	0.874
	MPA	72	0.123	0.302	0.173	0.293	0.014	0.940
	NEAT	74	0.174	0.138	**0.371**	**0.017**	−0.119	0.511

*^‡^Pearson’s correlation coefficient. *r*, Spearman’s correlation coefficient; SBP, systolic blood pressure; DBP, diastolic blood pressure; BMI, body mass index; HDL, high-density lipoprotein; LDL, low-density lipoprotein; MPA, moderate physical activity; NEAT, non-exercise activity thermogenesis score; VPA, vigorous physical activity. Bold values indicate significant at *p* < 0.05.*

**TABLE 3:** Multivariate regression models.

**Dependent variable**	**Predictors**	**Multivariate regression model**			**Adjusted *R*^2^ (model *p*-value)**
		**Coefficient**	**95% CI**	***p*-value**	
1/BMI	Sex	–0.00159	−0.00401, 0.00083	0.194	0.165 (< 0.001)
	Age	–0.00013	−0.00024, −0.00001	0.035	
	HbA_1c_	–0.00176	−0.00310, −0.00042	0.011	
	NEAT	0.00021	0.00008, 0.00034	0.001	

HbA_1c_	Sex	0.226	−0.104, 0.557	0.177	0.168 (< 0.001)
	CGMS use	–0.455	−0.826, −0.084	0.017	
	BMI	0.063	0.027, 0.099	< 0.001	
	NEAT	0.030	0.013, 0.047	< 0.001	

HbA_1c_	Sex	–0.0054	–0.3344.0.3236	0.974	0.130 (0.001)
	CGMS use	–0.3524	−0.7384, 0.0336	0.073	
	BMI	0.0533	0.0172, 0.0893	0.004	
	WORK	0.0002	0.0001, 0.0004	0.007	

HbA_1c_	Sex	0.1435	−0.2041, 0.4911	0.415	0.075 (0.021)
	CGMS use	–0.4442	−0.8406, −0.0479	0.028	
	BMI	0.0519	0.0136, 0.0902	0.009	
	LEISURE	–0.0002	−0.0008, 0.0004	0.443	

1/SBP	Sex	–0.00061	−0.00096, −0.00026	< 0.001	0.306 (< 0.001)
	BMI	–0.00010	−0.00014, −0.00006	< 0.001	
	NEAT	0.000002	−0.00002, 0.00002	0.830	

1/SBP	Sex	–0.00063	−0.00096, −0.00030	< 0.001	0.348 (< 0.001)
	BMI	–0.00011	−0.00015, −0.00007	< 0.001	
	MPA	–0.0000003	−0.00000, −0.00000	0.009	

1/SBP	Sex	–0.000603	−0.00094, −0.00027	< 0.001	0.349 (< 0.001)
	BMI	–0.000099	−0.00014, −0.00006	< 0.001	
	VPA	–0.0000003	−0.000001, −0.00000	0.012	

Cholesterol	Sex	–0.230	−0.565, 0.106	0.177	0.086 (0.027)
	BMI	0.00812	−0.026, 0.042	0.637	
	VPA	–0.00034	−0.001, −0.00009	0.009	

LDL	Sex	0.17110	−0.11030, 0.45250	0.229	0.093 (0.021)
	BMI	0.01087	−0.01783, 0.03956	0.452	
	VPA	–0.0003	−0.00052, −0.00001	0.005	

log(HDL)	Sex	–0.101	−0.154, −0.047	< 0.001	0.236 (< 0.001)
	BMI	–0.008	−0.014, −0.003	0.002	
	NEAT	–0.004	−0.006, −0.001	0.008	

log(HDL)	Sex	–0.078	−0.133, −0.022	0.007	0.134 (0.006)
	BMI	–0.007	−0.012, −0.001	0.021	
	MPA	–0.000008	−0.00004, 0.00002	0.607	

log(HDL)	Sex	–0.072	−0.124, −0.019	0.009	0.172 (0.001)
	BMI	–0.006	−0.012, −0.001	0.021	
	VPA	–0.000041	−0.00008, −0.000001	0.043	

*BMI, body mass index; CGMS, continuous glucose monitoring system; HDL, high-density lipoprotein; LDL, low-density lipoprotein; NEAT, non-exercise activity thermogenesis score; MPA, moderate physical activity; VPA, vigorous physical activity; LEISURE, total leisure PA; WORK, total work-related PA.*

### Glycemic Control

There was a significant positive correlation between NEAT and HbA_1__*c*_ when calculated for sample (*p* = 0.021) and sub-sample of men (*p* = 0.038), but not for women. Sub-analysis of people without usage of insulin pump or sensor technologies (*N* = 30) also showed positive correlation between HbA_1__*c*_ and NEAT (*r* = 0.49; *p* = 0.006). Additionally, a tendency toward significant positive correlation was obtained between HbA_1__*c*_ and VPA for the whole sample (*p* = 0.056). Furthermore, we obtained significant regression model explaining 16.8% of the variance of HbA_1__*c*_ predicted from sex, BMI, CGMS use, and NEAT. Higher BMI and NEAT levels were significantly associated with higher levels of HbA_1__*c*_, whereas in the same model the use of the CGMS significantly lowered HbA_1__*c*_ levels. On the contrary, when NEAT was substituted for MPA or VPA in the regression model, there were no significant associations with HbA_1__*c*_ (MPA *p* = 0.087, VPA *p* = 0.111). Total duration of work as assessed by GPAQ (moderate and vigorous) was significantly associated with HbA_1__*c*_ (*p* = 0.007) when adjusted for sex, BMI, and CGMS use, while such associations were not significant with total duration of recreation as assessed by GPAQ (*p* = 0.443). Multivariate regression models predicting frequency of hypoglycemia with sex, HbA_1__*c*_, and different types of PA were not significant, although there was a tendency toward significant negative correlation between VPA and frequency of hypoglycemia (*r* = −0.201, *p* = 0.050).

### Body Weight and Blood Pressure

Negative correlation between NEAT and BMI was obtained when performed for all participants (*r* = −0.352, *p* < 0.001) and for sub-sample of men (*r* = −0.411, *p* = 0.002), also similar significant associations were observed in the multivariate models when controlled for sex, HbA_1__c_, and age (Table 3). On the contrary, when NEAT was substituted by VPA and MPA, such associations with 1/BMI were not significant. There was a significant positive correlation between VPA and SBP when calculated for sample (*p* = 0.001) and sub-sample of women (*p* = 0.047), but not for men. SBP was positively associated with duration of MPA (*p* = 0.009) and VPA (*p* = 0.012), but not with NEAT score (*p* = 0.830) when controlled for sex and BMI, respectively. When the same multivariate models were applied for prediction of DBP, we failed to obtain significant models.

### Serum Lipids

Correlations of serum lipids concentrations and different types of PA were significant only between VPA and total cholesterol (*r* = −0.234, *p* = 0.046) and LDL cholesterol (*r* = −0.249, *p* = 0.034), respectively. Sub-analysis in men showed significant correlations between VPA and LDL cholesterol (*r* = −0.344, *p* = 0.028) and also between NEAT and triglycerides (*r* = 0.371, *p* = 0.017).

When adjusted for sex and BMI, NEAT and VPA explained 23.6 and 17.2% of logHDL variance, respectively. Higher levels of NEAT and VPA were significantly associated with lower levels of logHDL, resulting in association with higher HDL levels (after the outcome was transformed using the inverse logarithm). When applying the same independent variables for predicting total cholesterol and LDL levels, there was a significant association with sex, BMI, and VPA, explaining 8.6% of variance of total cholesterol and 9.3% of LDL levels. Also, regression models adjusted for sex and BMI showed that higher values of VPA were significantly associated with lower levels of total cholesterol (*p* = 0.009) and LDL (*p* = 0.005).

## Discussion

Physical activity has a potential to improve glucose metabolism in people with prediabetes and type 2 diabetes (Boniol et al., 2017). This potential is hindered by a challenge of maintaining blood glucose within range with insulin therapy, that is especially demanding for persons with type 1 diabetes, who often do not achieve appropriate chronic glucose control (McCarthy et al., 2016). To maintain appropriate glycemic profile around PA they often have to keep their blood glucose higher, reduce their insulin dose, and/or increase consumption of carbohydrates, especially when PA is not planned in advance (Francescato et al., 2015).

Results of our study failed to demonstrate benefit of LTPA on chronic glycemic control assessed by HbA_1__c_ in people with type 1 diabetes. Furthermore, we demonstrated a positive association of NEAT score and total duration of work activity assessed with GPAQ with chronic glycemic control, with no significant association with frequency of self-reported hypoglycemia.

Majority of people with type 1 diabetes do not meet the exercise levels as recommended by guidelines (McCarthy et al., 2016). Csizmadi et al. (2011) observed relatively low levels of leisure time activity among adult Canadian population, with occupational and household activity accounting for >80% of overall hours of daily PA. Low intensity PA of daily living constitutes about 20% of daily energy expenditure (Manohar et al., 2013) and is usually of longer duration, more frequent, and unpredictable compared to planned exercise. High absolute burden of PA of daily living and work on efforts for self-management of glycemia could be one of the factors for positive association with HbA_1__c_. Additionally, reducing inactivity by increasing the time spent walking/standing seems to be more effective in metabolic improvement than 1 h of physical exercise, when energy expenditure is kept constant (Duvivier et al., 2013).

Physical activity is frequently perceived as a significant risk of harm through hypoglycemia from perspective of people with diabetes (Brazeau et al., 2008). Fear of hypoglycemia is associated with higher HbA_1__c_ levels (Ahola et al., 2016) and could be one of the reasons for higher personal glycemic targets with insulin therapy while coping with daily glycemic imbalance associated with PA. HbA_1__c_ is not the only indicator of glycemic control, because dynamic in glycemic variability may not be reflected in this measure (Suh and Kim, 2015). Data on influence of chronic effects of exercise training on glucose variability are still scarce, even if it represents one of the most robust predictors of hypoglycemia (Klaprat et al., 2019). One of the promising options for managing degree of glucose variability associated with PA are emerging modern technologies (sensors with adequate accuracy for exercise, modern insulin pumps, and improvement in insulin analogs) (Houlder and Yardley, 2018), while their use and association with NEAT still requires further studies for clarification.

Large proportion of people with type 1 diabetes is obese or overweight (McCarthy et al., 2016), which is in line with results from our sample. Emerging evidence suggests that obesity contributes to insulin resistance, dyslipidemia, and cardiometabolic complications in type 1 diabetes, while prevalence of obesity increases with alarming rate in this population (Corbin et al., 2018). As expected from previous studies that emphasize the role of NEAT in preventing obesity (Ravussin, 2005), our results also suggest association of higher NEAT score with lower BMI in people with type 1 diabetes. Recent study by Du et al. (2019) shows a worrying trend in PA among US adults in years 2007–2016, with no improvement in duration of aerobic activity and statistically significant increase of sedentary behavior assessed by GPAQ. Recommendations on PA for weight management have traditionally been focused on leisure time activity, while other PA domains also offer many opportunities for interventions (Ravussin, 2005). Furthermore, there is a possible role of NEAT in behavioral compensation mechanisms after PA interventions that still awaits further clarification (Silva et al., 2018).

Predominant form of PA in people with type 2 diabetes are different domestic chores (Cloix et al., 2015). It is therefore not surprising that NEAT score seems to be associated with amelioration in insulin sensitivity, waist circumference, HDL, blood pressure, and the markers for atherosclerosis in people with type 2 diabetes without hypoglycemic therapy (Hamasaki et al., 2013). Limited research in type 1 diabetes suggests that interventions with exercise programs improve lipid levels, endothelial function, and insulin resistance but not blood pressure (Chimen et al., 2012). Exercise training does not significantly change resting SBP according to the recent meta-analysis (Ostman et al., 2018). Study of Bohn et al. (2015) did find an inverse association between LTPA and DBP without significant association with SBP. Surprisingly, we have observed a weak positive association of VPA and MPA with SBP in our sample when adjusted for sex and BMI, while we could not confirm such association with NEAT score. As expected from previous studies, our results also show positive association of NEAT score and VPA with HDL levels. Furthermore, we observed negative association of VPA with total and LDL cholesterol when adjusted for sex and BMI.

Because of relatively large sample size of our study, we could detect weak to moderate associations of sub-types of NEAT with different risk factors, which may have clinical implications in the population that has a known elevated risk for cardiovascular disease and sub-optimal risk factor control (McCarthy et al., 2016). Furthermore, frequent LTPA seems to reduce the risk of CVD events in people with type 1 diabetes in addition to traditional risk factors (Tikkanen-Dolenc et al., 2017). Exercise has also been shown to improve immune system function, body composition, physiological well-being, cardiovascular fitness, and late complications of diabetes in people with type 1 diabetes (Codella et al., 2017; Kluding et al., 2017). Further studies in type 1 diabetes are needed to evaluate the role of NEAT beyond traditional risk factors.

Our study has some important limitations, most important are cross-sectional design of the study and unspecific method (questionnaire) for estimating amount of NEAT. The validity of methods for measuring NEAT is still controversial. However, some authors believe that a questionnaire is one of the most useful and reliable methods of measuring NEAT at the moment (Hamasaki et al., 2013). Nevertheless, there is a need for use and further development of more precise methods for estimating NEAT (Manohar et al., 2013; Zecchin et al., 2013). Additional studies are also needed to exclude possible confounding variables (influence of diet, sociocultural background, and other factors) that may influence results of our analysis because of cross-sectional design of the study.

## Conclusion

Higher NEAT score is associated with lower body weight and higher HDL in adult people with type 1 diabetes, but may also present an additional burden for them with more challenging environment regarding glycemic control. New strategies for people with type 1 diabetes with higher levels of NEAT should be developed in the future to strengthen the protective effect of NEPA with combating modern sitting environment and also tackling the problem of the potentially greater glucose variability. Methods for precise estimation of NEAT should be further developed because of important role of this “silent” part of energy expenditure in people with type 1 diabetes, that is often overlooked in research and also in clinical practice.

## Data Availability Statement

The raw data supporting the conclusions of this manuscript will be made available by the authors, without undue reservation, to any qualified researcher.

## Ethics Statement

The studies involving human participants were reviewed and approved by the National Medical Ethics Committee of Slovenia (Ref. No. 0120-258/2017/4). The patients/participants provided their written informed consent to participate in this study.

## Author Contributions

IS, AZ, and VH created the study conception and design. AZ and IS were involved in the data collection. TK, IS, and AZ conducted the data analysis and wrote the manuscript. All authors were involved in drafting and revising the manuscript.

## Conflict of Interest

The authors declare that the research was conducted in the absence of any commercial or financial relationships that could be construed as a potential conflict of interest.

## Abbreviations

BMI: body mass index
CGMS: continuous glucose monitoring system
DBP: diastolic blood pressure
GPAQ: global physical activity questionnaire
HDL: high-density lipoprotein
LDL: low-density lipoprotein
LTPA: leisure time physical activity
MDI: multiple daily injections
MPA: moderate physical activity
NEAT: non-exercise activity thermogenesis
NEPA: non-exercise physical activity
PA: physical activity
SBP: systolic blood pressure
VPA: vigorous physical activity
